# Gynecological Notes

**Published:** 1894-10

**Authors:** T. Flavin

**Affiliations:** Demonstrator of Anatomy, Texas Medical College, Galveston


### GYNECOLOGICAL NOTES.

REPORTED BY T. FLAVIN, M. D.,
Demonstrator of Anatomy, Texas Medical College, Galveston.

HÆMATOMA OF OVARY.—George H Rohe, M. D., in *American Journal of Obstetrics*, June, 1893.

Dr. Rohe does not agree with the view that this condition is due to excessive hemorrhage into the Graafian follicle after rupture and escape of the ovule. Sometimes hæmatoma exists when no rupture has taken place, and corpora lutea frequently contain no blood. He thinks the view more rational that hæmatoma of the ovary is always to be regarded as a pathological formation having no connection with ovulation. He doubts whether any good purpose is served by the so-called "conservative" surgery which consists in extirpating the hæmotoma, stitching up the wound in the ovary, and dropping the organ back into the pelvis. Cases coming under his own observation have always presented either adhesions or displacements of the ovaries, and there are recognized indications for removal of the organs. Cites Dr. B. F. Black, "a careful and conservative gynecologist," in support of his views that ovaries affected with hæmatoma should be removed.

While admitting that the corpus luteum is an undoubted endothelial structure, Dr. Rohe thinks the statement of Dr. Foerster (*Am. Jour. Obst.* May, 1892) that "what was previously called a corpus luteum is invariably an endothelioma," is open to grave doubt. Then he quotes Dr. Foerster's statement as to the connection of the corpora lutea with the production of hæmatoma. In his own view, hæmatoma is probably due to some nutritional change in the ovary or its blood vessels, but what such change may be due to he leaves the pathologists to determine.

Dr. Rohe goes on to describe a case which he thinks shows that there is no essential difference between so-called ovarian apoplexy, where the entire ovary is converted into a blood cyst, varying in size from a billiard ball to a fœtal head, and those cases which present small collections of blood in the ovarian follicles and minute extravasations in the ovarian stroma.

An ovarian hæmatoma may rupture and give rise to pelvic hæmotocele; or bleeding may continue and patient die of hemorrhage. Peritonitis or sepsis is the most serious danger following rupture.

The diagnosis cannot be definitely made out before abdominal section; and the only rational proceeding is removal of the affected organ.

---

In the same number of the *Journal*, Dr. Angus MacKinnon describes an interesting case of vaginal hysterectomy for carcinoma uteri. He is led to do so by the belief that every case in which life has been saved or prolonged by the extirpation of a cancerous uterus should be reported in order to encourage its victims to submit to early operation.

Before entering into the details of his case, he points out that early operation is more urgent when the disease begins in the cervix than when it starts in the body of the uterus. In the former case the disease quickly spreads so as to involve the pelvic lymphatic glands, vagina, rectum and bladder; while in the latter, it may remain confined to the organ for a much longer period—phenomena which may possibly be accounted for by the more immediate vascular connection of the cervix with its surroundings and by the fact that the more fatal types of cancer rarely begin in the body of the uterus.

Among the points which made Dr. MacKinnon's case especially interesting was a vesico-vaginal fistula, which developed high up in the vagina during the second week after hysterectomy had been performed, and which obstinately refused to heal for thirteen months after the operation. All its features and characteristics pointed to cancerous affection, and in this view several physicians concurred. The interesting thing about it was that, whereas, it had previously resisted all therapeutic treatment, it yielded kindly to an ordinary operation for closure after the patient had undergone a severe attack of erysipelas, which started in the external genitals and spread therefrom over the whole body, lasting through five weeks. Now, the claim has frequently been made that erysipelas favorably modifies, or even cures cancer, and the history of this fistula, added to the fact that there has been no recurrence of the disease after two years, lends some color to the claim. In this connection it should be stated that the operator who performed the hysterectomy, Dr. H. C. Coe, of New York, predicted an early recurrence.

